# An improved deep learning network for image detection and its application in *Dendrobii caulis* decoction piece

**DOI:** 10.1038/s41598-024-63398-w

**Published:** 2024-06-12

**Authors:** Yonghu Chang, Dejin Zhou, Yongchuan Tang, Shuiping Ou, Sen Wang

**Affiliations:** 1 School of Medical Information Engineering, Zunyi Medical University, Zunyi, 563000 China; 2 School of Pharmacy, Zunyi Medical University, Zunyi, 563000 China; 3 School of Microelectronics, Northwestern Polytechnical University, Xi’an, 710072 China; 4 Department of Pharmacy, Affiliated Hospital of Zunyi Medical University, Zunyi, 563000 China

**Keywords:** *Dendrobii caulis* decoction piece, YOLOv5, Deep learning, Image processing, Computer science, Information technology, Software

## Abstract

In recent years, with the increasing demand for high-quality *Dendrobii caulis* decoction piece, the identification of *D. caulis* decoction piece species has become an urgent issue. However, the current methods are primarily designed for professional quality control and supervision. Therefore, ordinary consumers should not rely on these methods to assess the quality of products when making purchases. This research proposes a deep learning network called improved YOLOv5 for detecting different types of *D. caulis* decoction piece from images. In the main architecture of improved YOLOv5, we have designed the C2S module to replace the C3 module in YOLOv5, thereby enhancing the network’s feature extraction capability for dense and small targets. Additionally, we have introduced the Reparameterized Generalized Feature Pyramid Network (RepGFPN) module and Optimal Transport Assignment (OTA) operator to more effectively integrate the high-dimensional and low-dimensional features of the network. Furthermore, a new large-scale dataset of Dendrobium images has been established. Compared to other models with similar computational complexity, improved YOLOv5 achieves the highest detection accuracy, with an average mAP@.05 of 96.5%. It is computationally equivalent to YOLOv5 but surpasses YOLOv5 by 2 percentage points in terms of accuracy.

## Introduction

Dendrobium is a kind of commonly used traditional Chinese herbal medicine in the genus Dendrobium Sw. of the family Orchidaceae. Dendrobium is known as “longevity herb”, located in the first of the nine Chinese herbs. Dendrobium has a long history of application in China, and was first recorded in the Shennong Bencao Jing, which is the top grade of traditional Chinese medicine. Traditional Chinese medicine theory that Dendrobium taste sweet, slightly cold, with the benefit of the stomach, nourish the yin and clear the effect of heat, often used in the treatment of fever and fluid injury, dry mouth, thirst, lack of stomach yin, less food, dry vomiting, after the disease of false heat does not go away, yin deficiency and fire, bone vapour and labour heat, dark and unclear, sinew and bone impotence and softness; modern research has shown that Dendrobium is rich in polysaccharides, alkaloids, flavonoids, phenanthrenes, benzyl, volatile oil and amino acids and other ingredients, has the following properties Antioxidant, anti-inflammatory, lowering uric acid, lowering blood sugar, anti-tumour and immunomodulation and other pharmacological effects, in the treatment of cancer, hyperuricemia, diabetes and hepatic fibrosis liver function injury, enhance immunity, alleviate physical fatigue, skin anti-aging, anti-inflammatory repair, anti-radiation and so on, has a good application prospect^1–7^. There are many medicinal Dendrobium species, according to statistics, there are more than 1,500 species in the world, “Flora of China” recorded that there are about 78 species of Dendrobium spp. in China, which are mainly distributed in Guangxi, Guangdong, Guizhou, Yunnan, Fujian, Zhejiang, Jiangxi, Hunan, Anhui, etc. Among them, there are about 14 species are endemic to China, including Dendrobium hendianense, Dendrobium fanjingshanense, Dendrobium hainanense, etc.^8^. There are about 60 species of Dendrobium spp. in Yunnan Province, which is the region with the largest number of species of Dendrobium spp. in China. The Pharmacopoeia of the People’s Republic of China (2020 edition) includes several species such as Dendrobium hoshanense, Dendrobium chrysotoxum, Dendrobium fimbriatum, Dendrobium officinale, and Dendrobium nobileLindl. Among them, D. huoshanense is mainly produced in Huoshan County, Anhui Province in Dabie Mountain area, D.chrysotoxum is mainly produced in southern to western Yunnan, D. fimbriatum is mainly produced in southwestern to northwestern China, D. fimbriatum is mainly produced in Yunnan and Guizhou, and D.nobileLindl is mainly produced in Guizhou and Sichuan. At present, Dendrobium is used about 80,000 tons per year in China, mainly in the form of roughly processed slices of origin or slices produced in GMP-compliant workshops, which are widely used in products such as Chinese patent medicines, health care products, cosmetics, as well as in Chinese medicine for clinical use, folk health care and dietary therapy. Among them, D.hoshanense, D.chrysotoxum, D.fimbriatum, D.officinale, and D.nobileLindl. are circulated in the market in the form of primary processed products of agricultural products, because of their high price, the existence of shoddy and false situations in the market, which poses a great challenge to the authenticity of ordinary consumers.

Different species of Dendrobium are different in plant morphology, but their traits are similar after being processed into slices. The Pharmacopoeia of the People’s Republic of China (2020 edition) defines the traits of sliced Dendrobium species, and relevant national standards, industry standards, group standards and local standards also describe the traits of their slices. The description of the traits of this kind of medicinal herbs is a scientific and rigorous description of the terminology, which must have the expertise of botany, plant taxonomy, identification of herbs and other professionals can master and use, not applicable to non-professionals. Non-professionals have limited knowledge of Dendrobium, and when purchasing Dendrobium products, they are often faced with situations such as species being difficult to identify, substituting good for bad, and counterfeiting, which brings certain risks and economic losses to consumers.

At present, the main methods for identifying medicinal Dendrobium include morphological identification, microscopic identification, chromatographic identification, spectroscopic identification, molecular biology method identification, and recent rapid identification methods, such as near-infrared spectroscopic analysis^9–14^. These methods are mainly used for product quality testing and official supervision and arbitration testing, with strict requirements for methodological procedures, testing equipment and professional knowledge, and are not suitable for ordinary consumers to identify and judge the products when purchasing.

The motivation for the identification of *D. caulis* decoction piece detection and identification based on the improved YOLOv5 method designed in this paper is as follows.

- Ordinary consumers can only identify according to their own experience when purchasing, such as from the appearance of traits, color, texture, and smell or taste, etc. However, this kind of empirical judgments requires high knowledge of the consumers, the accuracy is not very high, and there are often cases of deviation.
- The image-based intelligent detection method of *D. caulis* decoction piece is an accurate, simple and fast identification method suitable for common consumers. Consumers only need to use their cell phones to take a photo of Dendrobium to be able to get the detection and classification results of Dendrobium in a visualized way. This can not only reduce the threshold of identification, but also save consumers’ time and energy.
- The image-based intelligent detection method of *D. caulis* decoction piece can minimize the possibility of substandard *D. caulis* decoction piece, standardize the market order of traditional Chinese medicine, and promote the healthy development of traditional Chinese medicine industry.

The contributions of this work are as follows. On the one hand, a YOLO-based recognition method for the detection of *D. caulis* decoction piece is proposed. On the other hand, a dataset of Dendrobium drinking slices detection and identification is established. The rest of the paper is organized as follows. Section 2 is a review of related research . In Section 3, a YOLO-based *D. caulis* decoction piece detection and recognition algorithm is proposed. The model training process and the result of ablation analysis are presented in Section 4. Section 5 is the conclusion of this work.

## Related work

A review of related researches in this work mainly focuses on two aspects. First, methods used to recognize the *D. caulis* decoction piece. Second, deep learning algorithms and their applications in object detection.

Although there is no method for target detection of *D. caulis* decoction piece, the researchers have utilized data augmentation and machine learning techniques to detect and classify plants. Traditional detection methods primarily rely on the extraction of shape and color features, making logical judgments based on the information extracted. Traditional target detection methods include Scale-Invariant Feature Transform (SIFT)^15^, Histogram of Oriented Gradients (HOG)^16^, Support Vector Machine (SVM)^17^, and Selective Search for object recognition^18^. Raphael et al. proposed a method for detecting fruits using hue information and color variation curvature, achieving a detection success rate of 78.8%^19^. Chunmei et al. extracted the Otsu feature from the image, then used the Otsu threshold algorithm for automatic threshold segmentation and extracted pixels representing the fruit, with an accuracy rate of over 95%^20^. Zhouzhou et al. improved the YOLOX model using techniques such as CSP Attention Block, SPPCSPC-F, and ASFF, resulting in a model named YOLOX-Nano, which achieved an mAP value of 84.08% for positioning^21^. Yuxiang et al. proposed a universal attention module (AGHRNet) capable of separating the background from the detected subject, which realized higher segmentation accuracy and smaller model parameters^22^. Mukhiddinov et al.^23^ presents a deep learning system for multiclass fruit and vegetable categorization based on an improved YOLOv4 model.but there is a certain loss of accuracy.Muhammad et al.^24^ proposing a novel DL-based methodology for the detection and classification of eight classes of weeds.but the detection rate is not that fast. Chowdhury et al.^25^ proposed a deep learning model based on EfficientNet and they used 18,161 tomato leaf images to classify tomato diseases. However, due to the emergence of the gradient vanishing problem, which makes the network difficult to train and difficult to converge. Liu et al.^26^ proposes a novel framework that combines hyperspectral imaging (HSI) and deep learning techniques for plant image classification.but does not perform well in small target scenarios. Teng et al.^27^ proposes propose a robust pest detection network based on RCNN.But it’s slow to detect.Wagle et al.^28^ proposed a CNN model with transfer learning from AlexNet to detect nine species of plants from the PlantVillage dataset.But it’s more computationally intensive.

Many computer vision algorithms based on CNN and deep learning have been proposed and hava been proven to be successful in the recognition and classification of real-world objects^29,30^.Some significant advancements in the field of computer vision, specifically in object detection, have been primarily focused on the RCNN^31^ series, YOLO^32^ series, and SSD^33^ series algorithms.R-CNN series includes R-CNN, Fast R-CNN^34^, Faster R-CNN^35^, and Mask R-CNN^36^. These methods achieve object detection through a process of region proposal extraction and region classification.YOLO series includes YOLO^37^, YOLOv2^38^, YOLOv3^39^, YOLOv4^40^, and so on. YOLO algorithms transform the object detection task into a regression problem and perform dense predictions directly on the image, enabling real-time object detection.SSD^33^ algorithm employs multiple scales of convolutional filters applied to feature maps at different levels to achieve object detection at various scales.It should be noted that the agricultural industry has turned to DL-based models to address these challenges. Deep learning approaches have achieved state-of-the-art results in tasks such as plant identification, fruit harvesting, and pest and disease control.

## Methods

### General research idea

**Figure 1:**
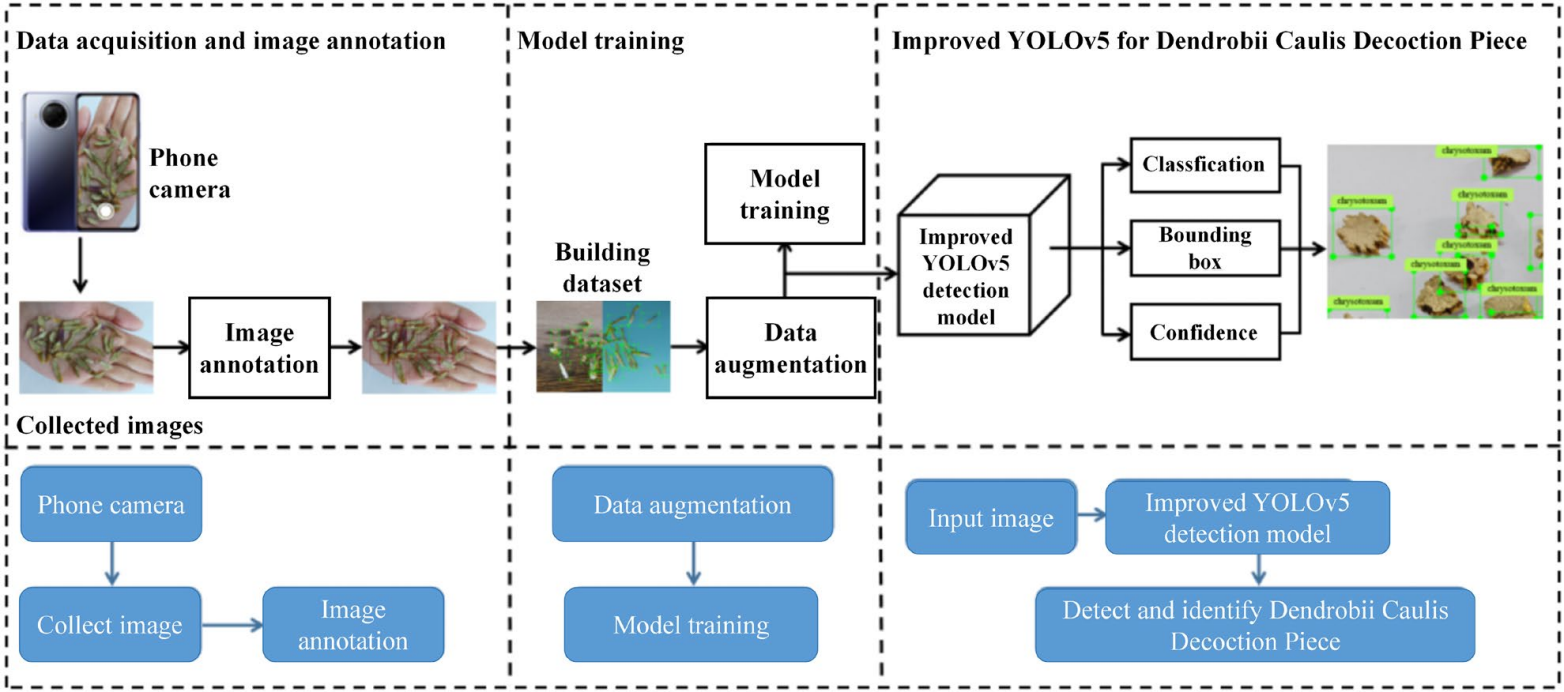
Framework of *D. caulis* decoction piece detection method based on deep learning.

Aiming at the application requirements of *D. caulis* decoction piece detection and recognition concerning real time and accuracy in actual Buying and selling scenarios, this paper proposes an improved YOLOv5 *D. caulis* decoction piece detection and recognition method for processing *D. caulis* decoction piece images collected by Phone camera. The overall framework is shown in Figure 1.

This study encompasses several key components, namely data collection, data annotation, data augmentation, and the development of an improved YOLOv5-based system for recognizing and classifying *D. caulis* decoction piece. Initially, photographs of *D. caulis* decoction piece were acquired using the smartphone’s camera, followed by the annotation process for these images. The collected images were then utilized to construct a comprehensive dataset, which underwent data augmentation techniques to enhance its diversity. Data augmentation methods were performed using an online approach and include random perspective, and HSV adjustments. Subsequently, the improved YOLOv5 model was deployed to accurately recognize and classify *D. caulis* decoction piece. Finally, the identified *D. caulis* decoction piece types were visually presented on the smartphone screen as images.

### YOLO-based meter detection and recognition algorithm

The recognition of *D. caulis* decoction piece imposes specific real-time performance requirements, necessitating the selection of mature object detection methods. Among these methods, the YOLO series, as a one-stage approach, exhibits faster detection speed compared to the two-stage RCNN series. Within the YOLO series, the YOLOv5 algorithm has emerged as a superior object detection algorithm due to its optimal trade-off between accuracy and speed. Compared to classic algorithms such as YOLOv3^39^ and YOLOv4^40^, YOLOv5^41^ boasts a more advanced network architecture that offers improved performance characteristics. In contrast to more recent and sophisticated models like YOLOv7^42^ and YOLOv8^43^, it employs a more lightweight architectural design, enabling it to achieve the desired performance on our dataset with a significantly reduced computational footprint. Therefore, this paper proposes an improved YOLOv5 algorithm for the identification of *D. caulis* decoction piece.

#### YOLOv5

**Figure 2:**
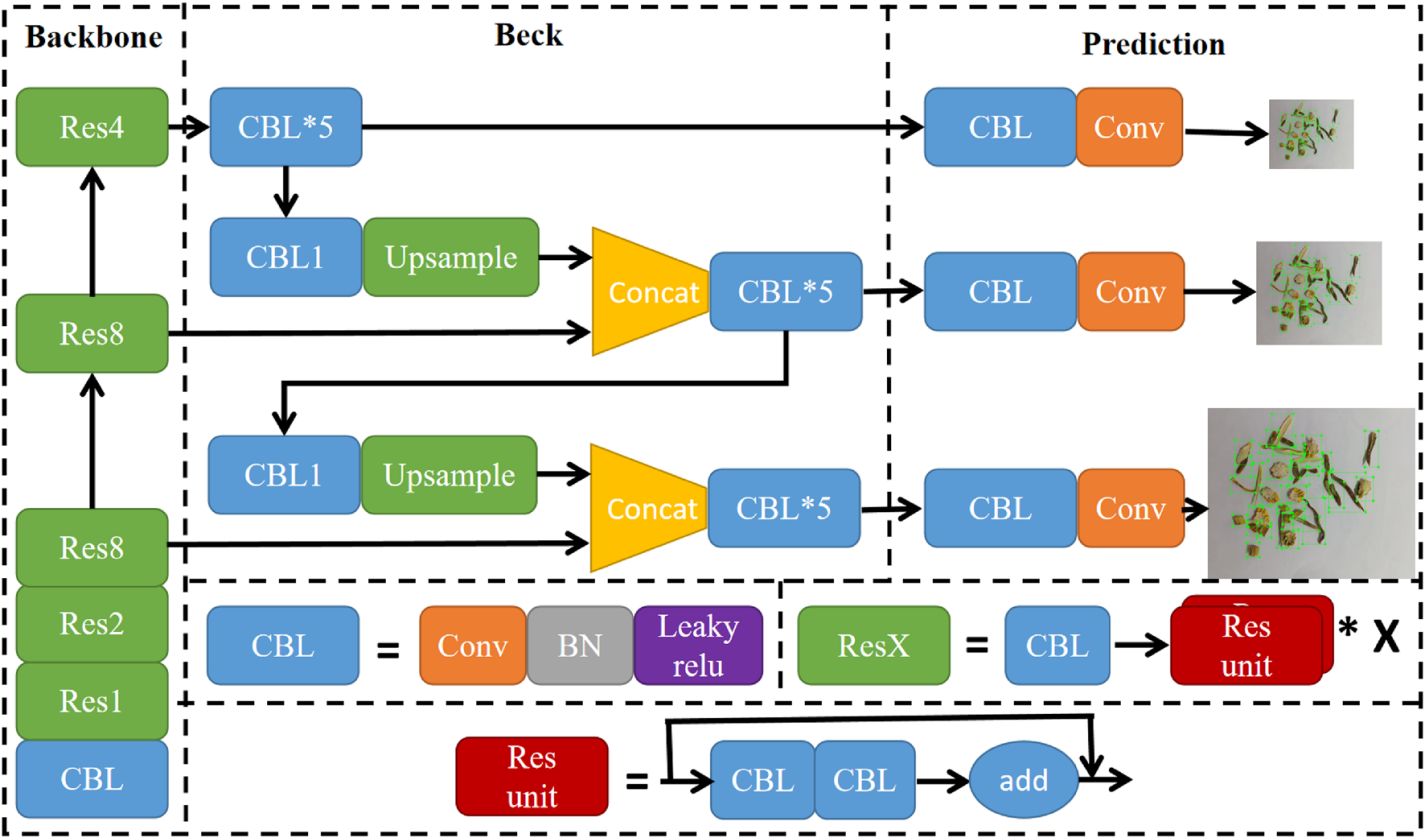
Framework of YOLOv5.

YOLOv5 employs CSPDarknet53 as its backbone network for extracting image features. It utilizes an FPN network to fuse three distinct output layer features from the backbone network. It use Mish activation function and the Focal Loss loss function to enhance the model’s performance.The overall structure of the YOLOv5 network is shown in Figure 2.

#### Improved YOLOv5

**Figure 3:**
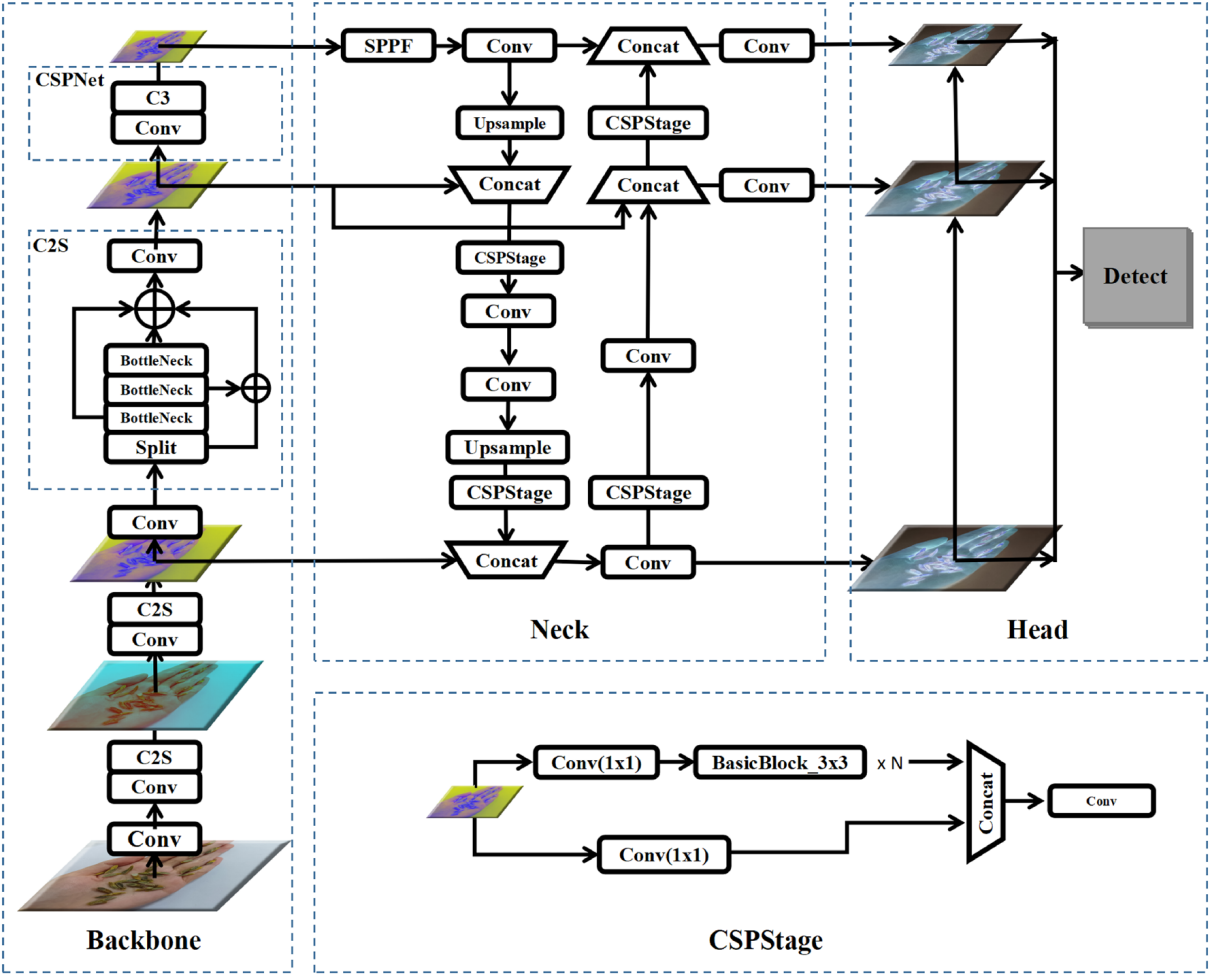
Improved YOLOv5 network structure.

Given that YOLOv5 is a lightweight network within the deep learning domain, efforts have been made to enhance network accuracy without significantly compromising network speed. The following improvements have been implemented to achieve this objective.The improved YOLOv5 network structure is shown in Figure 3.

- this paper designed a C2S module and add it to YOLOv5 Backbone. The C2S module interacts the feature maps of the current layer with deeper layers, leveraging semantic information from deeper layers to capture the position and detail information of small *D. caulis* targets. This enables the network to adapt to *D. caulis* targets with varying scales.
- This paper introduces the RepGFPN (Repeating Grouped Feature Pyramid Network) module to better utilize feature maps at different scales. The RepGFPN module divides the feature maps into multiple groups, performs feature fusion within each group, and then cascades the fusion results from different groups to achieve more effective feature fusion.
- The loss function incorporates optimal transport assignment, a dynamic label assignment method, into YOLOv5 Loss function to better handle class imbalance and varying target sizes.

Due to the small proportion of pixels occupied by small objects in images, the number of pixels in the feature maps obtained during the feature extraction process of convolutional neural networks gradually decreases after multiple downsampling operations. For instance, when using a stride of 16, a target region of size 32$\times$32 pixels reduces to only 2$\times$2 pixels in the feature map. This results in the loss of effective spatial information for detecting small objects, making it challenging to accurately detect them. Furthermore, as the network depth increases, the feature and positional information of small objects gradually diminish, further reducing the detection capability and localization accuracy of convolutional neural networks for small objects^44,45^. For tiny objects smaller than 10$\times$10 pixels, their target features become extremely weak or may even disappear after eight downsampling operations. Inspired by the YOLOv8, we made modifications to the C3 module by removing one convolutional block, adding multiple bottleneck layers, and introducing shortcut connections that combine shallow and deep features. This enhancement aims to strengthen the feature representation capability for small objects and improve their detection accuracy.

The feature pyramid network (FPN) is designed to aggregate features of different resolutions extracted from the backbone network, which has been proven to be a crucial and effective component in object detection^46–48^. FPN^46^ fuses feature maps from different levels through top-down feature propagation and lateral connections. However, it still suffers from the loss of features related to small objects. PAFPN^47^ introduces an additional bottom-up pathway aggregation network at the cost of increased computational complexity. BiFPN^48^ combines bottom-up and top-down feature propagation along with lateral connections to fuse feature maps at different levels and scales. This enables the model to extract object features at multiple scales and detect objects of varying sizes. Generalized-FPN (GFPN) has been proposed as the neck layer, achieving higher efficiency by effectively exchanging high-level semantic information and low-level spatial information. RepGFPN^49^, by flexibly controlling the depth and width of different channels using the CSPStage structure, achieves state-of-the-art performance with higher accuracy. We introduce the RepGFPN network into the neck layer of YOLOv5 to enhance performance. To reduce the computational overhead of RepGFPN without compromising the feature extraction capability of the backbone layer, we add a dimension reduction operation at the front end of the RepGFPN network. The features undergo the first convolutional operation, followed by halving the number of channels before entering the RepGFPN layer. This ultimately improves the network accuracy.

In the field of deep learning, particularly in the task of object detection, the YOLO (You Only Look Once) series of algorithms have garnered widespread attention due to their efficiency and accuracy. As an advanced variant within this series, YOLOv5’s loss function design has played a crucial role in enhancing the performance of the model. The loss function of YOLOv5 is a combination of multi-task losses, which simultaneously considers the classification, localization, and confidence of the targets. Specifically, the classification loss employs cross-entropy to measure the accuracy of class predictions. The localization loss uses Mean Squared Error (MSE) to gauge the discrepancy between the predicted bounding boxes and the actual bounding boxes. The confidence loss evaluates the model’s predictive confidence in the existence of the targets^50,51^. The formula is shown as follows:

1a $$\begin{aligned}&L_{total} = \sum _{i}^{N}\lambda _1L_{box}+\lambda _2L_{obj}+\lambda _3L_{cls} \end{aligned}$$

1b $$\begin{aligned}&= \sum _{i}^{N}\left( \lambda _1\sum _{j}^{B_i} L_{LCIoU}+\lambda _2\sum _{j}^{S_i\times S_i} L_{l_{obj_j}}+\lambda _3\sum _{i}^{B_i} L_{cls_j}\right) \end{aligned}$$

In the given formula, *N* refers to the number of detection layers, *B* represents the number of targets assigned to the prior boxes, and $S \times S$ denotes the number of grid cells into which the scale is divided. $L_{box}$ is the loss for bounding box regression, which is calculated for each object; $L_{obj}$ stands for the objectness loss, which is computed for each grid cell; $L_{cls}$ signifies the classification loss, also calculated for each object. $\lambda _1$, $\lambda _2$, and $\lambda _3$ are the weights for these three respective losses.

### Experiment

#### Data set construction

The images of *D. caulis* decoction piece were captured in Zunyi, China in November 2022. To ensure that the dataset covers a wide range of realistic lighting scenarios for *D. caulis* decoction piece identification, we considered different indoor lighting conditions as well as natural indoor lighting on sunny and cloudy days. To diversify the dataset, we included various background settings that are relevant to practical application scenarios, such as the palm of a hand, white paper, and different textured and colored tabletops. These images were captured using The cameras of the Xiaomi Note 11 at different angles and distances ranging from 0.3 to 0.5 meters. In total, 7,118 images of different *D. caulis* decoction piece were captured. Although the image sizes in the dataset are inconsistent, we applied a normalization step during deep neural network training to standardize all images to a fixed resolution of 640$\times$640. According to the Chinese Pharmacopoeia, there are five species of *D. caulis* decoction piece, including Dendrobium chrysotoxum, Dendrobium huoshanense, Dendrobium nobile Lindl., and Dendrobium nobile Lindl., all of which are included in our dataset. Each *D. caulis* decoction piece species was photographed individually as well as in combination with other species. To train a network with enhanced discriminative capabilities, we also captured a set of photographs containing a mixture of all different species of *D. caulis* decoction piece.

#### Evaluation metrics

For our dataset, each detected bounding box can be categorized into three scenarios. True Positives (TP) represent the detected bounding boxes whose intersection over union (defined as the ratio of intersection area to union area) with their corresponding ground truth bounding boxes is greater than 50%. False Positives (FP) represent the detected bounding boxes whose intersection over union is less than 50% with their corresponding ground truth bounding boxes. False Negatives (FN) represent the ground truth bounding boxes that are not covered by any detected bounding box.Precision reflects the accuracy of the model among all detected bounding boxes. It is defined as the ratio of the number of TP to the total number of detected bounding boxes.Recall reflects the model’s ability to cover all the ground truth bounding boxes.The formulas for precision (Prec), recall (Rec) are as follows:

2 $$\begin{aligned} Prec = \frac{TPs}{TPs+FPs} \end{aligned}$$

3 $$\begin{aligned} Recall = \frac{TPs}{TPs+FNs} \end{aligned}$$

The definition of mean Average Precision (mAP) is as follows:

4 $$\begin{aligned} mAP= \frac{1}{C} {\textstyle \sum _{C}^{i=1}Precision(i)} \end{aligned}$$

In the formula(4),C represents the total number of categories in the Shihu dataset. Prec(i) (represented by equation a(2)) denotes the precision for each category of Shihu.

#### Training details

We implemented improved YOLOv5 using PyTorch with Python version 3.8.0 and Torch version 1.13.1+cu1116. The training was performed on a single GPU (Nvidia RTX 3090). The improved YOLOv5 model was executed on a computer running Ubuntu 20.04 operating system with an Intel(R) Xeon(R) Silver 4210 CPU. The initial learning rate and learning rate scaling factor were both set to 0.01. Before the actual training, there was a warm-up period of 3 epochs, and the mini-batch size was set to 64. We utilized the Adam optimizer with a SGD momentum rate of 0.937, weight decay rate of 0.005, and a warm-up initial momentum rate of 0.8. The training process lasted for 300 epochs.

In order to prevent overfitting, we carefully considered the different angles of placement for *D. caulis* decoction piece and appropriately applied data augmentation algorithms during the model training process. The dataset comprises a sufficient number of images and was divided into 70% for training, 15% for validation, and 15% for testing. Specifically, the training set contains 4,990 images, the test set consists of 1,064 images, and the validation set comprises 1,064 images. Each set includes proportional representations of single herbs image, multiple herbs image, and mixed herbs image, with backgrounds consisting of palm, white paper, and various textures and colors of tabletops. To ensure that each training iteration receives a unique set of data augmentation effects, we have implemented online data augmentation, applying augmentations in real-time during the training process, rather than pre-applying augmentations and expanding the dataset beforehand. In addition, considering that end users may use the proposed algorithm to identify the Dendrobium under different lighting conditions, angles, and shooting distances, we applied two data augmentation techniques to the dataset: Random Perspective and HSV adjustments. For Random Perspective, we set the random rotation angle to range from -90 to +90 degrees, random translation along the X and Y axes with a magnitude of 0.1, random scaling with a factor of 0.8, and a random perspective transformation intensity of 0.001. As for HSV adjustments, we set the values of hsv_h to 0.015, hsv_s to 0.7, and hsv_v to 0.4. All image augmentation processes were implemented using the Albumentations library in Python. Following the convention, the network was trained for 300 epochs, during which the loss fluctuated within a small range, indicating convergence of the network.

#### Quantitative results

In order to demonstrate the effectiveness of improved YOLOv5, we compared it only with the detection models from the YOLO series. This is because currently, the YOLO series exhibits the best performance in various image object detection applications. The models we compared against YOLOv5^41^. Table 1 reports the quantitative results on our test images. Our improved YOLOv5 achieved the best performance in three out of the four metrics (Prec, mAP50, and mAP95) under the premise of relatively lower computational complexity.

**Table 1:** The quantitative comparison of several methods including YOLOv5 on the test dataset.

Methods	Prec	Rec	mAP@0.5	mAP@0.5:0.95
YOLOv3	0.908	0.892	0.933	0.535
YOLOv4	0.858	0.939	0.674	0.475
YOLOv5	0.922	0.925	0.945	0.590
Improved YOLOv5(ours)	0.928	0.960	0.965	0.621

In addition, to demonstrate the contribution of each module to the overall performance of improved YOLOv5, we individually integrated the OTA, RepGFPN, and C2S modules into YOLOv5 by replacing the corresponding components. Table 2 reports the quantitative results of the mentioned approaches. Both C2S, OTA, and RepGFPN outperformed YOLOv5 in terms of mAP50 and mAP50-95.

**Table 2:** The results of the peeling test of the network on the *D. caulis* decoction piece detection task.

Methods	Prec	Rec	mAP@0.5	mAP@0.5:0.95
YOLOv5	0.922	0.925	0.945	0.590
C2S	0.926	0.931	0.955	0593
OTA	0.923	0.954	0.963	0.625
RepGFPN	0.929	0.931	0.957	0.607

**Figure 4:**
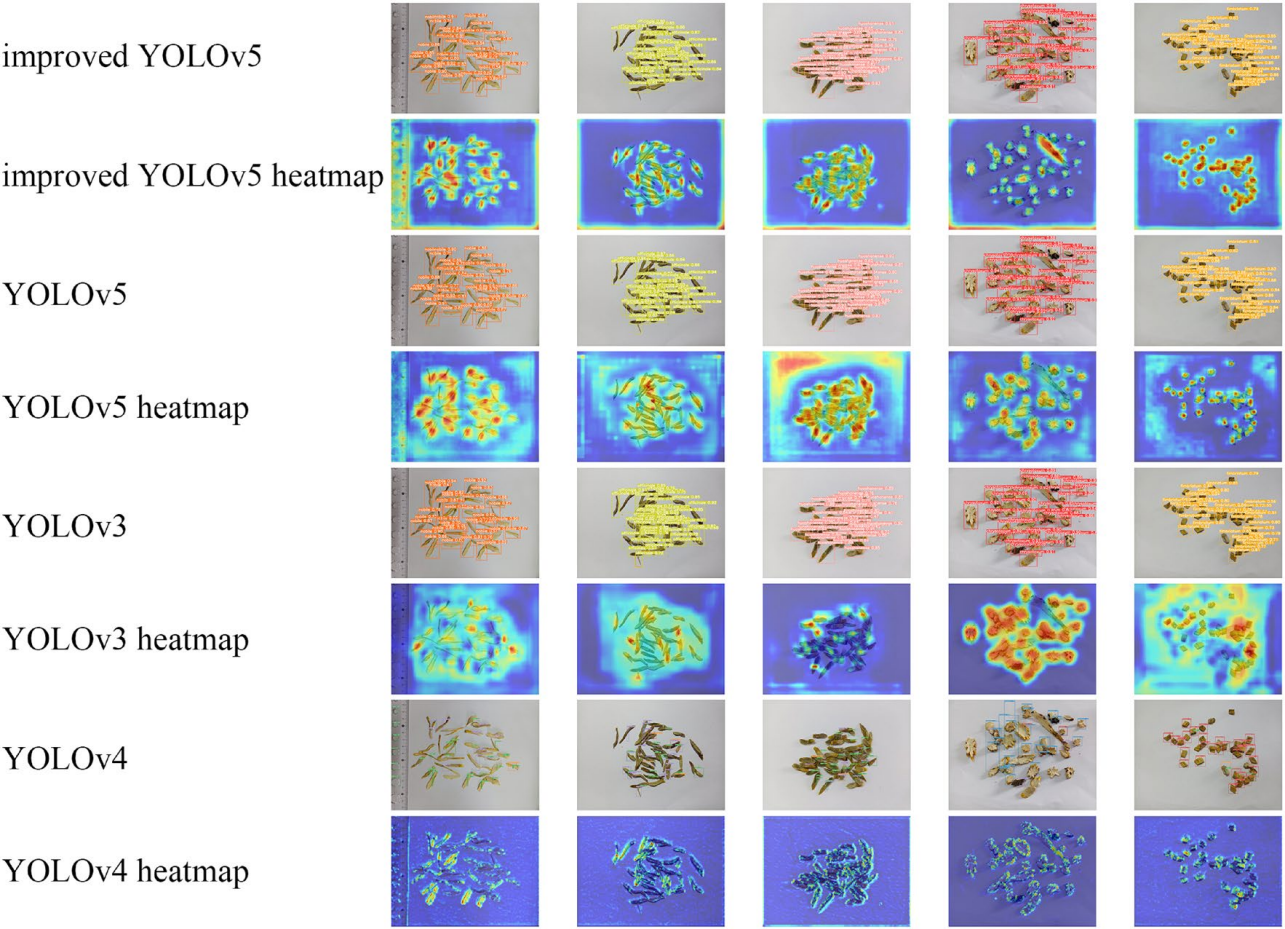
The visualization results of the model’s localization and classification.

we conducted an in-depth analysis of the performance of the YOLOv5, improved YOLOv5, YOLOv4, and YOLOv3 models on a our dataset and further observed the actual performance of the models through visualization techniques. To uncover the key regions that the models rely on for the identification and localization of objects of different categories within images, we employed the XGradCAM technology, specifically visualizing the last convolutional layer of the neck of each model. Utilizing heatmaps, we illustrated the areas of interest that the models focus on during the decision-making process, where red indicates high attention from the model, and blue signifies areas of relative neglect. The visualization results are shown in Figure 4.

Through comparative analysis, we found that YOLOv5 and its improved version performed excellently in detecting Dendrobium officinale slices, accurately identifying and classifying all samples. In contrast, YOLOv3 and YOLOv4 exhibited omissions during the detection process. Moreover, the YOLOv5 and its improved version demonstrated a significantly higher coverage of the regions of interest in the image compared to YOLOv3 and YOLOv4, indicating their superior capability in object localization. Particularly, in the improved YOLOv5, we observed a notable increase in the confidence of predictions compared to the standard YOLOv5, and the red areas in the heatmap corresponded more closely with the *D. caulis* decoction piece. This finding further confirms the effectiveness of the improved YOLOv5 in comprehending image features and enhancing detection accuracy.

### Ablation analysis

To demonstrate that YOLOv5 is the optimal combination of all the modules, we conducted a simple yet effective ablation analysis on the dataset. The results of all the ablation analyses are shown in Table 3. We compared the complete YOLOv5 model with the “YOLOv5,” “C2S+RepGFPN,” and “C2S+OTA” models using precision, recall, and mAP on the same dataset. The fully expanded YOLOv5 exhibited the best performance among the ablation comparisons.

**Table 3:** Experimental results of different combination models.

Methods	Prec	Rec	mAP@0.5	mAP@0.5:0.95
YOLOv5	0.922	0.925	0.945	0.590
Imporved YOLOv5(C2S+OTA)	0.922	0.925	0.955	0.620
Imporved YOLOv5(C2S+RepGFPN)	0.928	0.928	0.953	0.605
Imporved YOLOv5(C2S+GFPN+OTA)	**0.928**	**0.960**	**0.965**	**0.621**

*The best measures are in bold.

This paper focus on the research involving the use of deep learning models to achieve high-accuracy detection or recognition of different plants or fruits.Zhou et al.^42^ used a PSPNet to detect the endpoints of the dragon fruit, including dragon fruit segmentation and position,achieved an accuracy of around 95%. On the other hand, Huang et al.^52^ designed a deep learning network that combines UAV data collection, AI embedded device, and target detection algorithm to detection citrus with an accuracy of 93.32%. Likewise, Parico et al.^53–55^ used machine learning algorithms to accurately identify plants or fruits.In our work, improved YOLOv5 achieved an average mAP of 95.73% for multiple *D. caulis* decoction piece. the accuracy and mAP of the model are up, which is improved compared with the original baseline.Together with our improved YOLOv5, the above works disclose the popularity and the broad application prospects of machine learning and deep learning on fruit and plant detection.

### Ethical approval

The research was approved by the Guizhou Provincial Science and Technology Support Project (Program No. Qian Science Support [2018] 2804) including the permission to collect *D. caulis*. all the methods were carried out in accordance with relevant Institutional guidelines and regulations. Informed consent was obtained from all participants.

## Conclusions

This paper presents improved YOLOv5, a model for detecting and classifying *D. caulis* decoction piece, aiming to assist consumers unfamiliar with *D. caulis* decoction piece in quickly identifying the species using mobile devices such as smartphones. The network improves the capability of extracting features from small objects by introducing the C2S layer to replace the original C3 layer. It enhances the detection efficiency of the network by incorporating the OTA algorithm into the loss function. Additionally, the RepGFPN module is introduced in the feature fusion stage to better fuse shallow and deep features, achieving more effective feature fusion. We established a dataset and validated the effectiveness of the proposed method. The experiments demonstrate significant improvements in dense small object detection tasks compared to other state-of-the-art methods. The performance of the model can be attributed to the combination of learned shallow features and attention features, enabling our model to detect more small objects based on low resolution and weak features, thereby improving the recall rate of targets in dense and occluded scenes to some extent.

On one hand, for dense small objects, especially those with occlusions, our algorithm can improve their recall rate, but there are still undetected targets. Therefore, in future work, we will focus on addressing the detection of dense and occluded targets, such as using better post-processing mechanisms. On the other hand, compared to YOLOv5-Lite, we achieve better detection results but at a slightly slower speed and with higher computational complexity. Hence, we will further investigate methods to alleviate our approach and improve real-time detection speed. For example, depth-wise separable convolutions and lighter backbones can be explored as alternatives to the backbone of our method.

## Data Availability

Data and code are available from Sen Wang upon reasonable request.
